# Peptide Nucleic Acids as miRNA Target Protectors for the Treatment of Cystic Fibrosis

**DOI:** 10.3390/molecules22071144

**Published:** 2017-07-08

**Authors:** Federica Zarrilli, Felice Amato, Carmine Marco Morgillo, Brunella Pinto, Giuliano Santarpia, Nicola Borbone, Stefano D’Errico, Bruno Catalanotti, Gennaro Piccialli, Giuseppe Castaldo, Giorgia Oliviero

**Affiliations:** 1 Department of Biosciences and Territory, University of Molise, 86170 Isernia, Italy; zarrillif@gmail.com; 2 CEINGE–Advanced Biotechnologies Scarl, 80131 Napoli, Italy; felice.amato@unina.it (F.A.); giuseppe.castaldo@unina.it (G.C.); 3 Department of Molecular Medicine and Medical Biotechnologies, University of Naples Federico II, 80131 Napoli, Italy; 4 Department of Pharmacy, University of Naples Federico II, 80131 Napoli, Italy; carminemarco.morgillo@unina.it (C.M.M.); brunella.pinto87@gmail.com (B.P.); santarpiagiuliano@gmail.com (G.S.); nicola.borbone@unina.it (N.B.); stefano.derrico@unina.it (S.D.); brucatal@unina.it (B.C.); picciall@unina.it (G.P.)

**Keywords:** cystic fibrosis, CFTR, miRNA, miRNA target protectors, miR-509-3p, peptide nucleic acid, PNA

## Abstract

Cystic Fibrosis (CF) is one of the most common life shortening conditions in Caucasians. CF is caused by mutations in the CF Transmembrane Conductance Regulator (CFTR) gene which result in reduced or altered CFTR functionality. Several microRNAs (miRNAs) downregulate the expression of CFTR, thus causing or exacerbating the symptoms of CF. In this context, the design of anti-miRNA agents represents a valid functional tool, but its translation to the clinic might lead to unpredictable side effects because of the interference with the expression of other genes regulated by the same miRNAs. Herein, for the first time, is proposed the use of peptide nucleic acids (PNAs) to protect specific sequences in the 3’UTR (untranslated region) of the CFTR messenger RNA (mRNA) by action of miRNAs. Two PNAs (7 and 13 bases long) carrying the tetrapeptide Gly-SerP-SerP-Gly at their C-end, fully complementary to the 3’UTR sequence recognized by miR-509-3p, have been synthesized and the structural features of target PNA/RNA heteroduplexes have been investigated by spectroscopic and molecular dynamics studies. The co-transfection of the pLuc-CFTR-3´UTR vector with different combinations of PNAs, miR-509-3p, and controls in A549 cells demonstrated the ability of the longer PNA to rescue the luciferase activity by up to 70% of the control, thus supporting the use of suitable PNAs to counteract the reduction in the CFTR expression.

## 1. Introduction

One in every 3000 newborn Caucasians is affected by Cystic Fibrosis (CF), an autosomal recessive genetic disorder caused by mutations in a gene that encodes the cystic fibrosis transmembrane conductance regulator (CFTR) protein, a chloride-conducting transmembrane channel expressed in most epithelial and blood cells. Individuals who have inherited two mutated copies of the CFTR gene produce an altered CFTR protein (with decreased or absent CFTR chloride channel activity) that inhibits the flow of water and chloride ions across the cellular membranes and triggers the onset of clinical phenotypes characterized by an altered sweat test, pancreatic insufficiency, and pulmonary infections that gradually lead to respiratory insufficiency [1,2]. CF patients with at least one mutation that retains a residual channel activity could be treated with a strategy that aims to stabilize the CFTR messenger RNA (mRNA), thus increasing the amount of the protein and resulting in a net chloride flux increase [3]. Recently, it has been demonstrated that microRNAs (miRNAs) are also involved in the negative regulation of CFTR expression and consequently in the development and manifestations of CF lung disease [4,5,6,7,8,9,10]. MicroRNAs are evolutionarily conserved single-stranded non-coding RNAs, 18–25 nucleotides in length, that regulate the expression of specific genes at a post-transcriptional level, inhibiting the protein production [11,12,13]. In this context, the anti-miRNA strategy represents a valid tool within basic research and clinical applications. Indeed, our group used Peptide Nucleic Acids (PNAs) as anti-miRNA agents [14,15]. PNAs are mimics of DNA in which the sugar-phosphate backbone of the nucleic acid is replaced by a synthetic achiral peptide backbone. They emerged as one of the most promising candidates for gene therapeutics in antisense strategies and anti-miRNA approaches, showing a high affinity towards nucleic acid targets and generating very stable heteroduplex complexes. Moreover, PNAs and their complexes are not recognized by nucleases [16,17,18,19,20,21,22,23,24,25]. In a previous study, using an in vitro system based on the luciferase reporter system, we demonstrated that PNAs can inhibit miRNA activity and rescue the CFTR expression [14,15]. Although effective, the anti-miRNA approach suffers from a main limitation, i.e., it may interfere with other miRNA targets leading to unpredictable side effects. To overcome this problem and to counteract the effects of miRNAs binding to the CFTR mRNA [26], we propose the use of PNAs as miRNAs target protectors (TPs). This approach differs from the anti-miRNAs one because the PNAs block the miRNAs activity only for the specific gene of interest by competing with the miRNAs for the target mRNA. In the case of CF, the increased expression of the CFTR protein could lead to clinical benefit for all patients who retain residual activity of the CFTR channel. Furthermore, it has been shown that the expression of CFTR decreases during the development from embryo to adulthood. Therefore, an increase of the CFTR protein, and consequently of the flow of chloride through the membranes, could help those patients with at least one mutation that maintains some residual activity of the protein. In this study, we have synthesized and investigated, based on their ability to protect the CFTR mRNA, two PNA strands of different lengths (**1** and **2**, thirteen and seven bases in length, respectively, Table 1) that are fully complementary to the mRNA sequence recognized by the seed region of mir-509-3p. In addition, two other PNAs sharing the same base composition as **1** and **2**, but in random order (**3** and **4**, respectively, Table 1), have been designed and synthesized to confirm the sequence dependent activity of **1** and **2**. The negatively charged tetra-peptide Gly-SerP-SerP-Gly was installed at the C-end of all PNAs to improve their water solubility. The ability of **1** and **2** to recognize the mRNA was investigated by Circular Dichroism (CD) and UV studies on the corresponding PNA/DNA models and molecular modelling studies were performed to further confirm the structure and stability of the target PNA/mRNA complexes. We show that **1** proved effective in rescuing the luciferase expression by up to 70% of control in A549 cell culture model.

## 2. Results

### 2.1. Synthesis of PNAs ***1**–**4***

PNAs **1**–**4** (Table 1) were synthesized using the standard Fmoc-solid phase strategy on a 4-methylbenzhydrylamine (MBHA) resin. In the synthetic approach, the peptide tract was assembled at first, followed by the construction of the PNA portion. After the synthesis, the oligomers were detached from the support and lyophilized. The purification and analysis of the crude products were carried out using HPLC as outlined in the Materials and Methods. The electrospray mass spectrometry (ESI-MS) analyses confirmed the structure of the synthesized PNAs (Table 1).

### 2.2. Circular Dichroism (CD) and CD Melting Analyses

**Figure 1:**
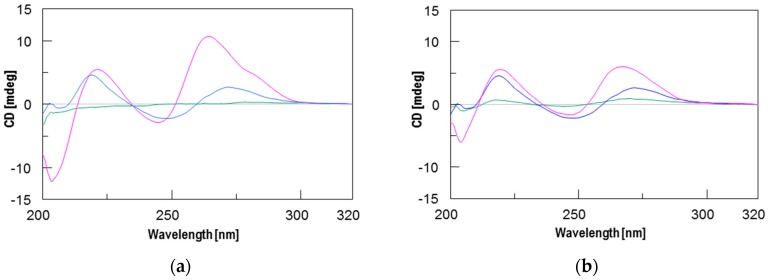
Circular dichroism **(**CD) spectra of: (**a**) PNA **1** (green), **ODN** (blue), and 1.5:1 **1**/**ODN** mixture (magenta); (**b**) PNA **2** (green), **ODN** (blue), and 1.5:1 **1**/ODN mixture (magenta). ODN is the DNA model sequence.

**Figure 2:**
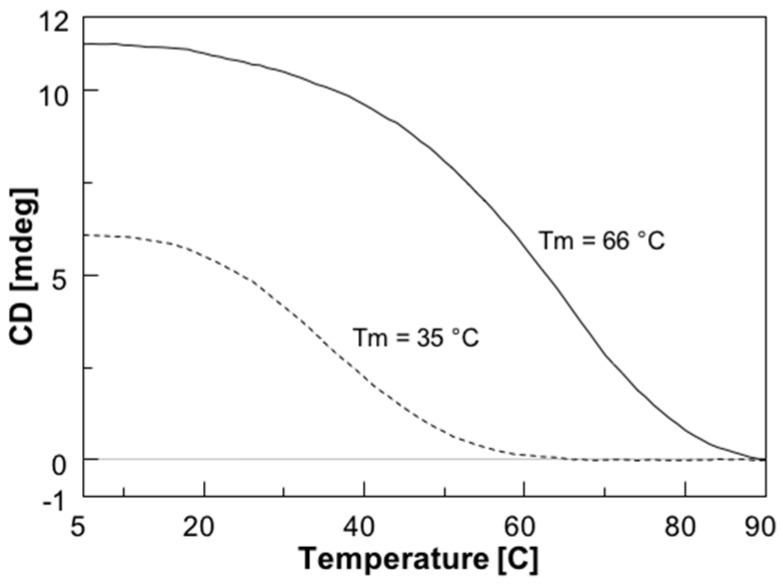
CD melting profiles of 1.5:1 PNA/**ODN** mixtures of **1** (solid line) and **2** (dashed line). The curves were obtained by monitoring the absorbance at 266 or 264 nm, respectively, for **1**/**ODN** or **2**/**ODN**, at a heating rate of 0.5 °C/min.

To assess the interaction between the new PNAs and the target sequence on the CFTR mRNA strand and the stability of the resulting PNA/RNA heteroduplexes, we used CD and CD melting analyses. However, because DNA is more resistant against nucleases and does not require any chemical modification for handling that could alter its recognition properties, the spectroscopic studies were performed using the corresponding 13-mer DNA model sequence (**ODN** in Table 1). Furthermore, considering the higher binding affinity of RNA strands relative to DNA strands towards complementary PNAs [27], the stability data obtained using the ODN model are probably underestimated. The formation of PNA/**ODN** heteroduplexes for **1** and **2** was assessed by recording the CD spectra of PNAs/**ODN** mixtures (1.5:1) in comparison with the CD spectra of PNAs and **ODN** alone. Each spectrum was recorded in phosphate buffered saline (PBS) buffer at 5 °C after the annealing procedure. Different from the CD spectra of PNAs and **ODN** alone, the spectra of both PNA/**ODN** mixtures showed the typical CD profile of antiparallel PNA/DNA heteroduplexes [28], characterized by maxima around 260 nm and 220 nm, and minima around 245 nm and 200 nm, thus confirming the formation of the target heteroduplexes **1**/**ODN** and **2**/**ODN** (Figure 1). The CD melting curves of both complexes are shown in Figure 2 and confirm, as expected, the greater stability of the longer **1**/**ODN** heteroduplex relative to that of the shorter one (Tm values of 66 °C and 35 °C, respectively). 

### 2.3. UV Studies

**Figure 3:**
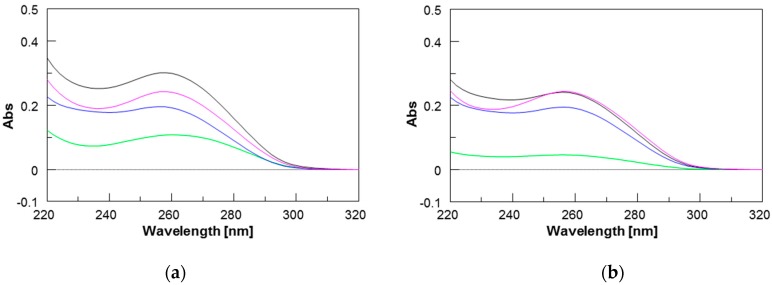
UV spectra of: (**a**) PNA **1** (green), **ODN** (blue), 1.5:1 **1**/**ODN** mixture (magenta), and the arithmetical sum of **1** and **ODN** (black); (**b**) PNA **1** (green), **ODN** (blue), 1.5:1 **1**/**ODN** mixture (magenta), and the arithmetical sum of **1** and **ODN** (black).

The formation of PNAs/**ODN** heteroduplexes was also monitored by UV spectroscopy by exploiting the hypochromic effect resulting from the formation of the heteroduplexes. The UV spectrum of each component alone and those of the mixtures are reported in Figure 3. All spectra were characterized by a maximum at around 260 nm, typical of nucleobases. The UV spectrum of the **1**/**ODN** complex (magenta curve in Figure 3a) showed lower values of absorbance than the arithmetic sum of each component alone (black curve in Figure 3a), thus confirming the occurrence of stacking interactions and the formation of the heteroduplex. In the case of the shorter PNA **2**, the UV spectrum of its complex with the **ODN** (violet curve in Figure 3b) showed almost the same values of absorbance as the arithmetic sum of its components, in agreement with the experimental lower stability of the shorter **2**/**ODN** heteroduplex. 

### 2.4. Molecular Modelling Studies

**Figure 4:**
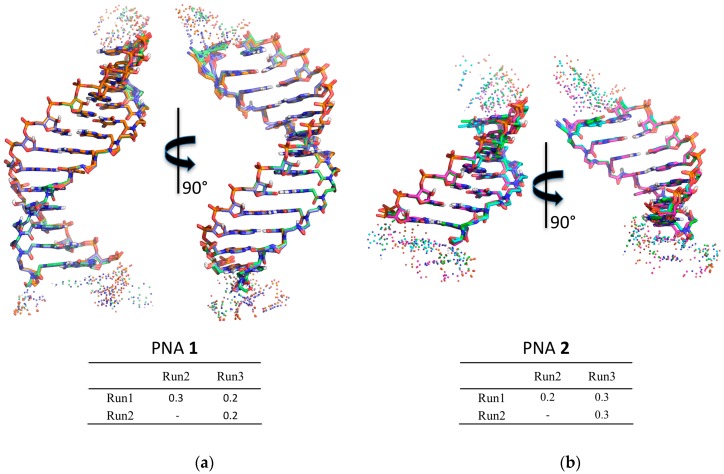
Superposition of average structures of the main represented cluster for **1**/**RNA** (**a**) and **2**/**RNA** (**b**), with relative root-mean-square deviation (RMSD) in Å calculated on heavy atoms. Duplex regions are represented in licorice, with carbons colored by molecular dynamics (MD) run (PNA **1**: Blue, Run 1; Orange, Run 2; Light green, Run 3. PNA **2**: Green, Run 1; Cyan, Run 2; Magenta, Run 3) and other atoms by standard convention (Oxygen in red, Nitrogen in blue, Phosphate in orange, and Hydrogen in white). **RNA** single strand flanking regions and the tetrapeptide Gly-SerP-SerP-Gly are represented in spheres coloured by MD run.

Molecular dynamics was used to check the structural features of the heteroduplexes **1**/**RNA** and **2**/**RNA** (Supplementary Materials, Table S1). The heteroduplexes were built starting from the NMR structure of the RNA(GAGUUC)/PNA(GAACTC) duplex (PDB-ID 176D) [29] as described in the Material and Methods section. The heteroduplexes were analyzed by means of three independent runs of molecular dynamics, for a total simulation time of 1200 ns for **1**/**RNA** system and of 900 ns for **2**/**RNA**. The chemical-physical properties of the systems, such as temperature, pressure, volume, density, and energy were fairly constant during the whole simulation for all the systems (data not shown). All the simulations were characterized by low root-mean-square deviation (RMSD) values and low fluctuations in the central duplex region (Supplementary Materials, Figure S1), thus indicating the presence of a stable structure. To evaluate the convergence between the independent runs of each system, a hierarchical clusterization of the molecular dynamics (MD) run was performed using Ambertools 15 [30]. The clusterization yielded in both cases one main cluster, accounting for more than 77% of frames for each run of PNA **1** and for more than 90% for PNA **2** runs (Supplementary Materials, Table S2 for details). Superposition of the average structure of the main cluster of each run showed very low RMSD values, thus indicating the convergence of the run to the same main structure (Figure 4; Supplementary Materials, Tables S3 and S4). 

To characterize the structural properties of the **1**/**RNA** and **2**/**RNA** heteroduplexes, local base pair step parameters (twist, roll, tilt, shift, slide, and rise), helical parameters (inclination), and the torsion angles of RNA and PNA monomers were calculated with the program Curves+ [31]. To limit the influence of the sterically less restricted terminal base pairs, the analysis was limited to the central 11 and 5 base pairs for the heteroduplexes formed by **1** and **2**, respectively. The results are reported in Table 2 and Table 3. All the heteroduplexes showed high similarity with the PNA/RNA heteroduplex experimental structures determined by NMR (PDB ID 176D [29]) and by XRAY (PDB ID 5EME and 5EMF) [32], with few noticeable deviations. In particular, **1**/**RNA** and **2**/**RNA** heteroduplexes showed lower values of twist and roll with respect to the experimentally determined structures, assuming values similar to those reported in previous MD studies [33].

The analysis of RNA torsion angles (Table 3) confirmed their close similarity with respect to the reference NMR PNA/RNA heteroduplex [29] and a strict consistence with the canonical A-RNA structure [34]. PNA angles, on the contrary, as previously reported [15,35,36,37,38], showed higher deviation and flexibility, particularly pronounced in the case of α and ε torsion angles, assuming values around −100° and 180°, respectively, in both PNA/RNA heteroduplexes (Supplementary Materials, Figure S1). 

**Figure 5:**
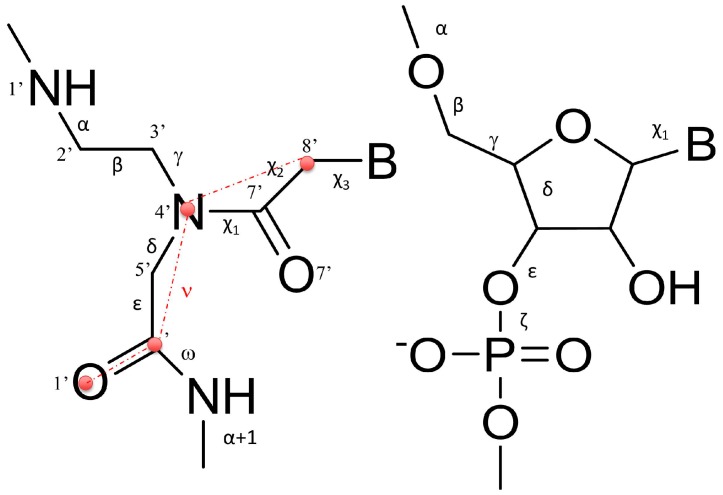
Schematic representation of the PNA (**left**) and RNA (**right**) backbones showing the definition of the torsion angles analyzed in Table 3. B stands for Base.

Interestingly, the analysis of the correlation between the torsion angle ε of the residue i and the torsion α_(i+1)_ of the subsequent base, showed the lack of consistent correlation between ε_i_ and α_(i+1)_. Moreover, to further explore structural features of PNAs/RNA heteroduplexes, we also evaluated the correlation between the pseudo-torsion angle ν_i_, defined as the angle between C8′-N4′-C′-O1′ (Figure 5, red circles), and the torsion angle α_(i+1)_ of the subsequent base, as a pointer of the orientation of the backbone carbonyl with respect to the strand terminus. The high Pearson correlation coefficients calculated revealed that there is a strong anti-correlation between the pseudo torsion angle ν_i_ and α_(i+1)_ of the subsequent base, thus revealing a prevalent orientation of the carbonyl group toward the N-terminus (Supplementary Materials, Table S5). A detailed discussion of the torsion angle behavior is given in Supplementary Materials (Text S1).

### 2.5. Biological Activity

**Figure 6:**
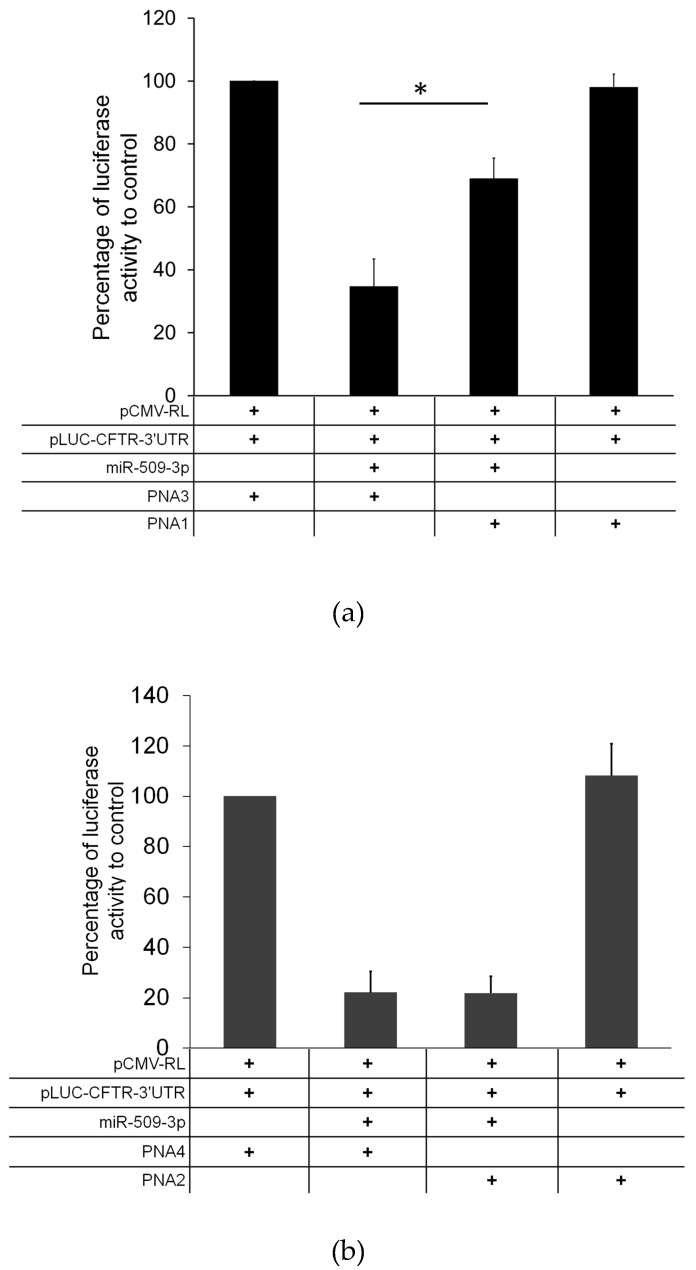
Effect of PNAs **1** (**a**) and **2** (**b**) on miR-509-3p activity. A significant rescue of the luciferase expression was observed using **1**. No significant activity was observed using **2**. * *p* values < 0.006.

Next, we tested the ability of PNAs **1** and **2** to block the inhibitor activity of miR-509-3p in a biological context. For this purpose, we tested the ability of the two PNA strands to rescue the reduction of luciferase activity induced by the transfection of miR-509-3p in A549 cells. Thus, we co-transfected different combinations of pLuc-CFTR-3′UTR (untranslated region) vector (a reporter luciferase construct sensitive to the miR-509-3p mimic action due to the presence of the 3′UTR of the CFTR gene), PNA strands, miR-509-3p mimic, and corresponding PNA scrambled controls. The co-transfection of miR-509-3p in the presence of the scrambled PNA **3** reduced luciferase expression leading to a residual activity up to 35%. The co-transfection of PNA **1** in the presence of miR-509-3p rescued the luciferase activity by up to 70% (Figure 6a). On the contrary, the addition of **1** alone did not produce any effect on the luciferase expression, thus indicating that **1** counteracts the inhibitory effects of miR509-3p by selectively competing for its binding on the 3′UTR of CFTR mRNA. Conversely, despite the spectroscopic and molecular dynamics evidence, the co-transfection of **2** in similar experimental conditions did not rescue the luciferase activity (Figure 6b).

## 3. Discussion

CFTR gene regulation by miRNAs plays an important role in lowering CFTR levels in CF patients. Therefore, much effort has been made to modulate miRNA control on CFTR. In a recent paper, we demonstrated that the activity of miR-509-3p miRNA, one of the miRNAs involved in the post-transcriptional regulation of the CFTR gene, could be inhibited using 14 or 7 bases long PNAs [14,15]. However, this type of strategy, as evidenced by some authors [26,39], has enormous off-target effects, because every miRNA regulates hundreds or thousands of mRNA targets. Alternatively, the use of miRNA Target Protectors (TPs) has been proposed as a promising option for the development of effective tools for the correction of CFTR expression in people with CF. In this context, we have here investigated the use of PNA strands as miRNA TPs. This strategy has the advantage of protecting the CFTR mRNA from binding with the miRNAs, which remain free to interact with their physiological mRNA targets. For this purpose, we have designed and synthesized two negatively charged PNAs, PNAs **1** and **2** (Table 1), functionalized at the C-end with the negatively charged tetrapeptide Gly-SerP-SerP-Gly to improve the water solubility and cellular uptake. The chemico-physical characterization of PNA/nucleic acid heterocomplexes was performed through CD, CD melting and UV experiments. To ease the nucleic acid handling during the CD and UV investigations, all spectroscopic investigations were performed using the 3’UTR mRNA DNA mimic (**ODN** in Table 1) rather than the corresponding RNA strand. This approach allowed us to avoid the use of a nucleases-resistant chemically-modified RNA strand, which could have affected the binding affinity with the studied PNAs. Circular dichroism measurements are useful in the characterization of the secondary structure of nucleic acids and in the study of hybridization events. In this case, the CD studies confirmed the capability of PNAs **1** and **2** to bind the target ODN strand and form the corresponding heteroduplexes, whose melting temperatures were determined by derivatization of the resulting CD melting curves (64 °C and 35 °C, respectively; Figure 3). In addition, the comparison of the UV spectra shown in Figure 4 revealed a lower absorbance than the arithmetic sum of each component alone for the **1**/ODN heteroduplex. This confirms the occurrence of strong stacking interactions between the DNA strand and **1**. The same behavior was not observed for the UV spectra of **2**/ODN complex (Figure 5), even if the formation of the heteroduplex was supported by CD and molecular dynamics studies. A possible explanation for this behavior could be found in the lower number of bases involved in the Watson-Crick (W-C) base pairing for the **2**/ODN complex relative to the total number of bases in the two strands (PNA + ODN). Furthermore, the MD simulations of **2**/RNA complex showed high fluctuations for the terminal base pairs (Supplementary Materials, Figure S1C), indicating that only four or five bases could be involved in stable W-C interactions in the heteroduplex. Finally, we also performed MD simulations of **1**/RNA and **2**/RNA (1200 and 900 ns, respectively) to characterize the structural features of these heteroduplexes conjugated to a negatively charged tetrapeptide tail. The analysis of helicoidal parameters and torsion angles (Table 2 and Table 3) revealed that both MD derived PNA/RNA heteroduplexes showed similar structural features. PNA **1**- and **2**-containing helices were slightly unwound and less bent with respect to the experimentally determined structures of PNA/RNA heteroduplexes, as indicated by the lower twist values. On the other side, the helices appeared slightly less bent than both NMR and XRAY derived structures because of lower roll values. The roll parameter, indeed, indicated the degree of rotation with respect to the main helical axis, and, therefore, higher roll values are associated to higher perturbation of the coplanarity of bases, inducing the bending of the helix. The analysis of torsion angles of RNA and PNA backbones confirmed structural features very similar to those of reference experimental structures. RNA strand torsion angles closely resembled those found in the reference PNA/RNA NMR structure and in the canonical A-RNA structure. On the other side, as previously reported [15,35,36,37,38], the PNA strand showed higher flexibility, particularly in torsion angles α and ε, that may assume two sets of values −100° and 100° and −20° and 180°, respectively (Supplementary Materials, Figure S2 and Text S1). Taken together, these results indicated that the negatively charged tetrapeptide slightly affects the structural features of the heteroduplexes with respect to experimentally determined PNA/RNA heteroduplexes. 

The ability of PNAs **1** and **2** to protect the miR-509-3p target site in the 3′UTR of CFTR gene was evaluated by testing the effects on the expression of the luciferase gene cloned upstream. The results have shown clearly that the transfection of the 7 bases long PNA **2** in A549 cells co-transfected with miR-509-3p did not increase significantly the luciferase gene expression (Figure 6b), whereas the co-transfection with the longer PNA **1** reduced the miR-509-3p gene inhibition by up to 70% (Figure 6a). These results are apparently in contradiction with the results of our previous paper on the efficacy of 7 and 14 bases long anti-miRNA PNAs to rescue the luciferase expression in the presence of miR-509-3p [15] (which showed almost the same potency for the two PNAs). A possible explanation for the observed different behavior may be found in the different mechanism of action of the two approaches. The anti-miRNA mechanism is based on the competition of PNAs with the miRNAs for the mRNA target, whereas the activity of miRNA TPs is indeed the result of the formation of heteroduplexes between PNAs and the target mRNA. In the latter case, the length of PNAs directly correlates with the binding affinity for the target mRNA, due to the increasing number of base pairs involved in longer duplexes. On the other side, the anti-miRNA approach requires that the PNAs interact with miRNAs integrated in the RNA-induced silencing complexes, which result from the association of miRNAs with proteins of the argonaute family [40]. Hence, the interaction of longer PNAs with the miRNA-protein complex rather than with the free miRNA could be disfavored relative to the interaction of shorter PNAs.

## 4. Materials and Methods

### 4.1. General Methods

All reagents and solvents were obtained from commercial sources and used without further purification. Phosphoramidites for DNA syntheses were purchased from Glen Research (Sterling, VA, USA). The ODNs were assembled by using the PerSeptive Biosystems Expedite DNA/RNA 8909 synthesizer using phosphoramidite chemistry. Peptide nucleic acid monomers were purchased from Link technologies (Bellshill, Lanarkshire, UK). Fmoc-L-Ser[PO(OBzl)OH]-OH was purchased from Iris Biotech GmbH (Marktredwitz, Germany). Fmoc-Gly-OH and the MBHA resin (1% divinylbenzene, 200–400 mesh, 0.5 mmol/g loading) were purchased from Sigma-Aldrich (Saint Louis, MO, USA). The reactions on solid phase were performed using ISOLUTE® single fritted reservoirs (SG), 20 μm PE (polyethylene), equipped with tube caps and luer tip caps Biotage (Uppsala, Sweden) which were shaken in a Multi-reax vibrating shaker Heidolph (Schwabach, Germany). High performance liquid chromatography (HPLC) analyses and purifications were carried out on a Jasco UP-2075 Plus pump equipped with a Jasco (Easton, MD, USA) UV-2075 Plus UV detector using a 4.8 × 150 mm C-18 reverse-phase column (particle size 5 μm) eluted with a linear gradient of CH_3_CN containing 0.1% (*v/v*) trifluoroacetic acid (TFA) in H_2_O containing 0.1% (*v/v*) TFA (from 0 to 100% in 45 min, flow 1.2 mL/min). UV spectra were recorded on a Jasco V-530 spectrophotometer (Jasco). CD spectra were performed on a Jasco 1500 spectropolarimeter (Jasco) equipped with a Jasco PTC-348 WI Peltier-type temperature controller in a 0.1 cm path length cuvette. ESI-MS experiments were performed on an Applied Biosystems (Warrington, Cheshire, UK) 4000 QTRAP mass spectrometer in positive ion electrospray mode, dissolving the compounds in H_2_O containing 0.1% (*v/v*) formic acid.

### 4.2. DNA Synthesis and Analysis

The oligonucleotide 5′GAAGCACCAATCA3′ was synthesized using solid phase β-cyanoethyl phosphoramidite chemistry. After the synthesis, the oligomers were detached from the support and deprotected by treatment with concentrated aqueous ammonia at 55 °C for 12 h. The combined filtrates and washings were concentrated under reduced pressure, redissolved in H_2_O, and analyzed and purified by HPLC on a Macherey Nagel (Düren, Germany) Nucleogel SAX column 1000-8/46 using buffer A: 20 mM NaH_2_PO_4_ aqueous solution, pH 7.0, containing 20% (*v/v*) CH_3_CN; buffer B: 1 M NaCl, 20 mM NaH_2_PO_4_ aqueous solution, pH 7.0, containing 20% (*v/v*) CH_3_CN; a linear gradient from 0% to 100% B in 30 min and flow rate 1.2 mL/min were used. The oligomers were collected and successively desalted by Sep-Pak cartridges (C18). The isolated oligomers were >99% pure (NMR). The ODN concentration was determined spectrophotometrically at λ = 260 nm and 90 °C, using the molar extinction coefficient ε = 200.9 cm^−1^·mM^−1^ calculated by the nearest neighbor mode.

### 4.3. PNA Synthesis and Analysis

PNA sequences were synthesized using the Fmoc-solid-phase strategy. Fifty milligrams of MBHA resin (0.5 mmol/g), after swelling in CH_2_Cl_2_ for 30 min and DMF washings, was treated with a solution of 20% piperidine in DMF for 10 min. After washings in DMF (×5), the resin was reacted with Fmoc-Gly (5 eq. in NMP 0.2 M), 1-[Bis(dimethylamino)methylene]-1*H*-1,2,3-triazolo[4,5-b]pyridinium 3-oxid hexafluorophosphate (HATU) (5 eq. in DMF 0.2 M) and *N*,*N*-Diisopropylethylamine (DIPEA) (5 eq.)/lutidine (7.5 eq.) for 45 min at room temperature. Couplings of Fmoc-L-Ser[PO(OBzl)OH]-OH were achieved using the following conditions: Fmoc-Ser monomer (8 eq. in NMP 0.2 M), HATU (8 eq. in DMF 0.4 M), and DIPEA (8 eq.)/lutidine (12 eq.) for 15 h at room temperature. After the serine couplings, a further glycine residue was attached on the N-terminal of the serine tract following the previously described coupling with the glycine monomer. PNA monomers were reacted using the following conditions: monomer building block (10 eq. in NMP 0.2 M), HATU (10 eq. in DMF 0.2 M), and DIPEA (10 eq.)/lutidine (15 eq.), 45 min at room temperature. After each coupling step, capping with Ac_2_O in the presence of pyridine was performed for 20 min at r.t. Fmoc group was removed by a treatment with a 5% 1,8-Diazabicyclo[5,4,0]undec-7-ene (DBU) in DMF solution (5 min). In the case of Fmoc-Ser amino acids, the basic treatment was prolonged (20 min). At the end of synthetic cycles, the PNAs were cleaved from the solid support by treatment with TFA/anisole/ethanedithiol (9:1:1; *v/v/v*) for 4 h and the products were precipitated with cold diethyl ether. The precipitates were recovered by centrifugation, washed twice with diethyl ether, dissolved in water, and finally lyophilized. PNAs **3** and **4**, chosen as the negative control and bearing the same functionalization of PNAs **1** and **2**, were synthesized using the same standard Fmoc-solid-phase strategy. The PNAs were obtained with a 48–50% overall yield (94–95% medium yield for each coupling as estimated by Fmoc spectrophotometric measurements). The crude sample was purified by semipreparative reverse phase HPLC (see General methods). The collected fractions were lyophilized and the final pure product was characterized by ESI-MS (positive mode): ESI-MS (*m/z*) calcd. for PNAs **1** and **3** 4003.4; found [M + 3H]^3+^ 1335.5, [M + 4H]^4+^ 1002.0; PNAs **2** and **4** 2411.8; found [M + 2H]^2+^ 1206.9, [M + 3H]^3+^ 805.0. The amount of each PNA sample dissolved in pure water was estimated by quantitative UV at 90 °C using the following molar extinction coefficients: PNAs **1** and **3** ε = 126.5 mL·µmol^−1^·cm^−1^; PNAs **2** and **4** ε = 75.2 mL·µmol^−1^·cm^−1^ and DNA ε = 134.2 mL·µmol^−1^ ·cm^−1^. 

### 4.4. Preparation of DNA/PNA Heteroduplexes (Annealing Procedure)

The PNA/ODN heteroduplexes (1.5:1) were obtained by dissolving the mixture of the samples at the concentration of 2.0 × 10^−5^ M in 100 mM PBS and by heating the solution to 90 °C for 5 min and then slowly cooling to room temperature over 12 h. 

### 4.5. UV

The UV spectra were recorded with a Jasco V-530 UV spectrophotometer, in 100 mM PBS buffer at the concentration of 20 μM. They were recorded at 20 °C (λ = 220–310 nm, 400 nm/min scanning speed, 2.0 nm bandwidth).

### 4.6. CD and CD Melting Studies

The CD spectra were recorded with a Jasco 1500 spectropolarimeter equipped with a Peltier-type temperature controller (PTC-348 WI) in a 0.1 cm cuvette, in 100 mM PBS buffer at the concentration of 20 μM. They were recorded at 5 °C (λ = 220–310 nm, 200 nm/min scanning speed, 2.0 nm bandwidth) and averaged over three repetitions. A buffer baseline was subtracted from the CD spectra and the spectra were normalized to have zero at 320 nm. Thermal denaturation experiments were also carried out in the temperature range of 5–90 °C by monitoring the CD values at 266 nm for PNA **1**/ODN and at 264 nm for PNA **2**/ODN at a heating rate of 1.0 °C/min.

### 4.7. Molecular Dynamics (MD) Simulations

The initial structures were built following the same procedure described in [15], starting from the NMR structure of the RNA(GAGUUC)/PNA(GAACTC) duplex (PDB-ID 176D) [28]. The correct sequence was obtained by mutating the bases using the X3DNA software [41]. Each heteroduplex was built including three flanking bases on both the 3′ and 5′ ends of the RNA segment (Supplementary Materials, Table S1). Thermalization of the duplex and production of MD trajectories were obtained using Amber 15 suite [30]. The leap module of Ambertools 15 was used to perform the parameterization of the systems, using the ff14SB force field (AMBER99SB and frcmod.ff14SB for peptide + ff99bsc0_chiOL3 for RNA) [42,43,44], and the Sanders et al. parameters for PNA [37], whereas the parameters for serine phosphate were taken from [45]. TIP3P water molecules were added with at least a minimum spacing of 14.0 Å between the edge of the box and the molecules. Na^+^ counterions were added to neutralize the system. The system was geometrically minimized in three steps: (i) optimization of hydrogen atoms with 2000 steps of steepest descent algorithm and 8000 steps of conjugate gradient algorithm, (ii) optimization of water molecules and counterions with 2000 steps of steepest descent and 18,000 steps of conjugate gradient, and (iii) optimization of the whole system with 2000 steps of steepest descent and 8000 steps of conjugate gradient. The equilibration of the system was performed using the protocol described in [15]. Briefly: (i) the system was thermalized in 240 ps, raising the temperature from 10 K to 298 K with a time step of 1 fs, and applying inter- and intra-strand constraints of 20 kcal·mol^−1^·Å^−2^ in order to preserve, respectively, the W-C base pairs and the torsional angles of the RNA sugar; ii) the constraints were gradually removed from 20 kcal·mol^−1^·Å^−2^ to 0.1 kcal·mol^−1^·Å^−2^ in 240 ps at constant pressure (1 bar) and temperature (298 K). Finally, an equilibration step of 500 ps was run without constrains. Production runs were performed using a time step of 2 fs. The SHAKE algorithm was used for all hydrogen atoms in conjunction with periodic boundary conditions at constant pressure and temperature. Particle mesh Ewald was used for the treatment of long range electrostatic interactions, and a cut-off of 9 Å was used for non-bonded interactions. Each system was studied by means of 300 ns MD simulation runs in triplicate with random seeding for the initial velocities. In order to investigate more deeply the stability of the PNA **1**/RNA system, we extended one run to 600 ns, for a total of 1200 ns for the PNA **1**/RNA and 900 ns for the PNA **2**/RNA. The analysis of the structure was carried out using the software Curves+ [31]. The visualization of the trajectories and related snapshots were performed with Pymol [46] andVMD [47], while the trajectory post-processing analysis was performed using Ambertools15 [30]. Cluster analysis was performed through a hierarchical agglomerative (bottom-up) approach using a root-mean-square (RMS) metric comparing the heavy atoms in the central duplex base-pairs. Correlation analysis was performed calculating the Pearson correlation coefficient on torsion angles sampled each 0.01 ns.

### 4.8. Cell Line, Construct, and Transfections

A549 human lung carcinoma cells were purchased from ATCC (Manassas, VA, USA). The cells were maintained in Dulbecco’s modified Eagle’s medium (Gibco Invitrogen, North Andover, MA, USA) with 10% heat inactivated fetal bovine serum (HyClone Laboratories, South Logan, UT, USA) without the addition of antibiotics. A Luciferase construct bearing the 3′UTR of the CFTR gene was used as the miR-509-3p sensitive reporter system. The transfection of the A549 cells with miRNA-mimics (Qiagen, Hilden, Germany) or PNAs was performed with the Attractene Transfection Reagent (Qiagen). Briefly, the cells seeded in 96-well plates were cotransfected with the luciferase reporter construct, miR-509-3p mimic and the PNAs. The luciferase activity level was measured 24 h after transfection using the Dual-Glo Luciferase Assay System (Promega Corporation, Madison, WI, USA). The EnSpire Multimode Plate Reader (Perkim Elmer, Waltham, MA, USA) was used for the luminescence assay using 96-multiwell black plates. The relative reporter activity was obtained by normalization to the Renilla luciferase activity.

## 5. Conclusions

In this paper, we have proposed for the first time the use of PNAs as miRNA target protectors to increase the expression of CFTR in CF. The negatively charged PNAs **1** and **2,** conveniently modified at their C-ends and fully complementary to the 3’UTR region of the CFTR mRNA recognized by the seed region of miR-509-3p, were synthesized and characterized. To demonstrate the sequence dependent activity of **1** and **2**, two other PNAs (**3** and **4**, Table 1), containing scrambled sequences of **1** and **2**, respectively, were designed and synthesized. Spectroscopic data confirmed the ability of **1** and **2** to bind their complementary ODN target by forming stable PNA/ODN heteroduplexes. The structural features of **1**/RNA and **2**/RNA heteroduplexes were also determined through molecular dynamics simulations. The results indicated that the presence of the negatively charged tetra peptide at the C-end of **1** and **2** slightly affected the structural features of the resulting PNA/RNA heteroduplexes (with respect to the experimentally determined PNA/RNA heteroduplexes). Biological studies show that the PNA molecules are suitable to counteract the action of miR-509-3p (in this case) as miRNA target protectors. These data confirm, once again, that a mRNA-targeted approach of a gene to increase the expression of the protein could be a good therapeutic strategy (that, of course, could be also used in other monogenic diseases). Moreover, the PNAs, showing a high affinity towards nucleic acid targets and forming stable heteroduplex complexes, represent excellent candidates to be used for this approach. Furthermore, this type of approach has the advantage of being relatively mutation-independent. For this reason, it would be sufficient to increase the expression of a protein, even if mutated, to exceed the threshold of minimal activity needed to obtain an optimal clinical phenotype.

## Figures and Tables

**Table 1:** Sequences of the cystic fibrosis (CF) transmembrane conductance regulator (CFTR) messenger RNA (mRNA) and of peptide nucleic acids (PNAs) **1**–**4**. UTR = untranslated region.

Name	Sequence ^1,2^
3’UTR of CFTR mRNA (**RNA**)	G-A-A-G-A-A-G-C-A-C-C-A-A-U-C-A-U-G-A
DNA model sequence (**ODN**)	G-A-A-G-C-A-C-C-A-A-T-C-A
PNA **1** (C → N)	*G-S(P)-S(P)-G*–c-t-t-c-g-t-g-g-t-t-a-g-t
PNA **2** (C → N)	*G-S(P)-S(P)-G*–g-g-t-t-a-g-t
PNA **3** (C → N)	*G-S(P)-S(P)-G*–c-a-g-t-t-g-t-c-t-g-t-g-t
PNA **4** (C → N)	*G-S(P)-S(P)-G*–t-t-g-g-a-g-t

^1^ The tetrapeptide tail at PNAs C-end is written in italics. ^2^ Target of miR-509-3p seed region is in red.

**Table 2:** Helicoidal parameters of the average structure of the more representative cluster of **1/RNA** and **2/RNA** MD simulations. Standard deviations are reported in brackets.

Name	Shift	Slide	Rise	Tilt	Roll	Twist
**1/RNA**	−0.7 (0.2)	−1.9 (0.2)	3.3 (0.2)	1.0 (1.4)	2.4 (1.9)	23.6 (1.7)
**2/RNA**	−0.9 (0.3)	−1.7 (0.2)	3.3 (0.1)	1.3 (1.5)	3.9 (1.9)	22.6 (1.3)
PNA/RNA (NMR) ^1^	0.3 (0.3)	−1.4 (0.6)	3.2 (0.4)	−2.8 (1.3)	4.6 (4.3)	29.4 (3.8)
PNA/RNA (MD) ^2^	-	-	-	-	-	24
PNA/RNA (X-RAY) ^3^	−0.8 (0.4)	−2.1 (0.3)	3.3 (0.1)	0.1 (1.3)	6.9 (3.5)	25.0 (1.6)
A-RNA ^4^	-	-	2.8	-	-	32.7

^1^ Calculated on the average structure of PDB 176D, from [29]; ^2^ from [33]; ^3^ calculated on the average structure of PDBs 5EME and 5EMF, from [32]; ^4^ from [34].

**Table 3:** Average torsion angle values of the average structure of the more representative cluster of **1/RNA** and **2/RNA** MD simulations. Standard deviations are reported in brackets. The torsion angle definition is given in Figure 5.

**Torsional PNA Angles**									
**Name**	**α**	**β**	**γ**	**δ**	**ε**	**ω**	**χ_1_**	**χ_2_**	**χ_3_**
**1/RNA**	–120 (46.0)	78 (10.6)	71 (2.5)	95 (1.8)	–177 (42.9)	–146 (10.0)	–2 (1.2)	–164 (2.2)	84 (2.8)
**2/RNA**	–136 (43.2)	76 (4.1)	79 (3.9)	96 (2.0)	–162 (56.6)	–172 (10.6)	–3 (1.6)	–152 (18.0)	77 (12.6)
PNA/RNA (NMR) ^1^	160 (10.1)	68 (2.4)	81 (5.0)	59 (16.1)	–104 (6.7)	–177 (3.2)	12 (3.3)	–118 (8.6)	49 (10.2)
PNA/RNA (XRAY) ^2^	–177 (83.5)	68 (8.8)	71 (7.5)	95 (5.2)	–123 (81.0)	–178 (10.5)	5 (5.1)	–173 (4.0)	82 (6.6)
**Torsional RNA Angles**									
**Name**	**α**	**β**	**γ**	**δ**	**ε**	**ζ**	**χ**		
**1/RNA**	–77 (1.5)	172 (1.7)	68 (1.5)	79 (0.9)	–162 (2.7)	–72 (2.9)	–160 (3.1)
**2/RNA**	–78 (1.6)	173 (1.9)	67 (1.6)	79 (1.3)	–164 (2.7)	–73 (2.4)	–156 (2.8)
PNA/RNA (NMR) ^1^	–68 (6.7)	171 (13.8)	58 (1.2)	79 (3.6)	–149 (23.8)	–73 (11.6)	–168 (4.4)
PNA/RNA (X-RAY) ^2^	–80 (4.9)	177 (5.4)	63 (4.9)	79 (6.2)	–158 (5.5)	–71 (6.6)	–164 (4.9)
A-RNA ^3^	–79 (43.5)	172 (19.9)	64 (34.7)	80 (9.53)	–152 (17.6)	–76 (24.7)	–162 (9.76)

^1^ Calculated on the average structure of PDB 176D, from Ref. [29]; ^2^ calculated on the average structure of PDBs 5EME and 5EMF, from Ref. [32]; ^3^ from Ref. [34].

## Supplementary Materials

Supplementary materials are available online.
