# Supplementary material for: Peptide Nucleic Acids as miRNA Target Protectors for the Treatment of Cystic Fibrosis
Source: Molecules. 2017 Jul 8;22(7):1144. doi: 10.3390/molecules22071144 (PMC6152032; doi:10.3390/molecules22071144)
Supplement: Supplementary file 1 [file molecules-22-01144-s001.pdf]

Peptide Nucleic Acids as miRNA Target Protectors for the Treatment of Cystic Fibrosis

Federica Zarrilli [1,2], Felice Amato [2,3], Carmine M. Morgillo [4], Brunella Pinto [4], Giuliano Santarpia [4], Nicola Borbone [4], Stefano D'Errico [4], Bruno Catalanotti [4], Gennaro Piccialli [4], Giuseppe Castaldo [2,3] and Giorgia Oliviero [3,*]

[1] Department of Biosciences and Territory, University of Molise, Isernia, Italy
[2] CEINGE – Advanced Biotechnologies Scarl, Napoli, Italy
[3] Department of Molecular Medicine and Medical Biotechnologies, University of Naples Federico II, Napoli, Italy
[4] Department of Pharmacy, University of Naples Federico II, Napoli, Italy
* Correspondence: golivier@unina.it; Tel.: +39-081-679-896

Text S1: Correlation between torsion angles in the PNA strands.

The analysis of PNA torsion angles performed with Curves + showed a high degree of deviation and flexibility, and particularly pronounced in the case of α and ε torsion angles. Indeed, in all MD simulations, two main average values were found, for α (-100 ° and 100 °) and ε (-20 ° and 180 °) (Figure S2). The analysis of distribution of torsion angles revealed that in our simulation 80% of values ranged around -100 °, in both **1**/RNA and **2**/RNA heteroduplexes, while ε assumed preferentially values around 180 ° (Figure S1). To check whether the discrete values assumed by these torsion angles were correlated, the Pearson correlation coefficient was calculated on the torsion angle ε of the residue i and the torsion $\alpha_{(i+1)}$ of the subsequent base, sampled on 0.1 ns interval. Indeed, it has been previously reported that these angles were correlated in PNA containing duplexes [S-1].

The results, reported in Table S5, showed that for both **1**/RNA and **2**/RNA heteroduplexes the Pearson correlation coefficients between ε_i and $\alpha_{(i+1)}$ are all below 0.3, suggesting the lack of consistent correlation.

To further explore structural features of PNA/RNA heteroduplexes, was also analysed the correlation between the pseudo torsion ν_i and the torsion angle α of the subsequent base. The torsion angle ν, defined as the angle between C8′-N4′-C′-O1′ (Main text, Figure 8, red circles), has been proposed as a pointer of the orientation of the backbone carbonyl with respect to the strand terminus [S-2]. The high Pearson correlation coefficients calculated revealed that there is a strong anti-correlation between the pseudo torsion angle ν_i and $\alpha_{(i+1)}$ of the subsequent base (Main text, Table 3).

The correlation found between negative values of $\alpha_{(i+1)}$ and positive values of ν_i in both **1**/RNA and **2**/RNA duplexes determines the orientation of the backbone carbonyl towards the N-terminus. Accordingly, the measure of the distance between the two carbonyl oxygens in each PNA base resulted in the range 3.4 Å - 4.1 Å, in good agreement with previously reported data [S-2].

References

S-1. He, W.; Hatcher, E.; Balaeff, A.; Beratan, D.N.; Gil, R.R.; Madrid, M.; Achim, C. Solution structure of a peptide nucleic acid duplex from NMR data: features and limitations. *J. Am. Chem. Soc.* **2008**, 130(40), 13264-13273.

S-2. Soliva, R.; Sherer, E.; Luque, F.J.; Laughton, C.A.; Orozco, M. Molecular dynamics simulations of PNA·DNA and PNA·RNA duplexes in aqueous solution. *J. Am. Chem. Soc.* **2000**, *122(25)*, 5997–6008.

Table S1. Heteroduplexes systems modelled and analysed by MD simulations. The tetrapeptide tail at PNA C-end is reported in italics characters.

<table>
<tr><td>PNA 1 /RNA</td><td>G_1-A_2-A_3-G_4-A_5-A_6-G_7-C_8-A_9-C_{10}-C_{11}-A_{12}-A_{13}-U_{14}-C_{15}-A_{16}-U_{17}-G_{18}-A_{19}
| | | | | | | | | | | | |
$\mathit{G_{36}}$-$\mathit{S(P)_{35}}$-$\mathit{S(P)_{34}}$-$\mathit{G_{33}}$-$\mathbf{c}_{32}$-$\mathbf{t}_{31}$-$\mathbf{t}_{30}$-$\mathbf{c}_{29}$-$\mathbf{g}_{28}$-$\mathbf{t}_{27}$-$\mathbf{g}_{26}$-$\mathbf{g}_{25}$-$\mathbf{t}_{24}$-$\mathbf{t}_{23}$-$\mathbf{a}_{22}$-$\mathbf{g}_{21}$-$\mathbf{t}_{20}$</td></tr>
<tr><td>PNA 2 /RNA</td><td>G_1-C_2-A_3-C_4-C_5-A_6-A_7-U_8-C_9-A_{10}-U_{11}-G_{12}-A_{13}
| | | | | | |
$\mathit{G_{36}}$-$\mathit{S(P)_{35}}$-$\mathit{S(P)_{34}}$-$\mathit{G_{33}}$--$\mathbf{g}_{20}$-$\mathbf{g}_{19}$-$\mathbf{t}_{18}$-$\mathbf{t}_{17}$-$\mathbf{a}_{16}$-$\mathbf{g}_{15}$-$\mathbf{t}_{14}$</td></tr>
</table>

Table S2. Output of the hierarchical clusterization performed with Ambertools 15 using a RMS metric comparing the heavy atoms in the central duplex base-pairs. Only clusters with population higher than 0.1% were reported.

```
---------------------------------------------------------------
#Run1 PNA 1
#Cluster   Frames     Frac  AvgDist    Stdev Centroid AvgCDist
       0     2315    0.772    1.941    0.518     1285    3.029
       1      481    0.160    1.947    0.551     2693    3.085
       2      127    0.042    1.862    0.485     1748    2.916
       3       74    0.025    1.788    0.388     1473    3.783
       4        2    0.001    2.385    0.000     2355    3.798

#Run2 PNA 1
#Cluster   Frames     Frac  AvgDist    Stdev Centroid AvgCDist
       0     6263    0.973    1.881    0.557     3612    2.836
       1      171    0.027    1.871    0.557     5365    3.676

#Run3 PNA 1
#Cluster   Frames     Frac  AvgDist    Stdev Centroid AvgCDist
       0      408    0.816    3.498    0.828      207    5.008
       1       49    0.098    3.407    0.740      161    4.975
       2       37    0.074    3.555    0.773      143    5.312
       3        5    0.010    3.327    0.808      167    5.616
       4        1    0.002    0.000    0.000      444    6.192
---------------------------------------------------------------
#Run1 PNA 2
#Cluster   Frames     Frac  AvgDist    Stdev Centroid AvgCDist
       0     3063    0.974    1.099    0.237       96    1.538
       1       31    0.010    1.062    0.207     1431    1.796
       2       29    0.009    1.246    0.226     2317    1.937
       3       20    0.006    1.211    0.251      246    1.676

#Run2 PNA 2
#Cluster   Frames     Frac  AvgDist    Stdev Centroid AvgCDist
       0     2937    0.987    1.144    0.243     1030    1.739
       1       29    0.010    1.221    0.256      884    1.889
       2        5    0.002    1.205    0.157     1409    1.906
       3        3    0.001    1.385    0.284     1044    1.996
       4        3    0.001    1.283    0.328     1248    1.898

#Run3 PNA 2
#Cluster   Frames     Frac  AvgDist    Stdev Centroid AvgCDist
       0     2991    0.997    1.198    0.263     1163    1.802
       1        5    0.002    1.543    0.271     2172    1.967
       2        2    0.001    0.933    0.000     2143    1.996
---------------------------------------------------------------
```

Table S3. RMSD in Å between the average structures of clusters with population higher than 5% obtained from the MD simulation of **1**/RNA heteroduplex.

#Cluster	run2_0	run3_0
run1_0	0.118	0.234
run2_0	-	0.242

Table S4: RMSD in Å between the average structures of clusters with population higher than 5% obtained from the MD simulation of **2**/RNA heteroduplex.

#Cluster	run1_1	run2_0	run3_0	run3_1	run3_2
run1_0	2.207	0.305	0.216	1.902	1.793
run1_1	-	2.268	2.240	1.504	2.880
run2_0	-	-	0.196	1.806	1.703
run3_0	-	-	-	1.893	1.797
run3_1	-	-	-	-	2.013

Table S5: Pearson correlation coefficients between torsion angles ε_i and $\alpha_{(i+1)}$ and between the pseudo torsion angle ν_i and the torsion angle $\alpha_{(i+1)}$. Indexes were calculated on the central bases of **1**/RNA or **2**/RNA heteroduplexes, on the basis of 0.1 ns sampling of torsion angles. Definition of torsion angles is given in Main text, Figure 8.

	Pearson ε_i:$\alpha_{(i+1)}$			Pearson ν_i:$\alpha_{(i+1)}$		
**1**	Run1	Run2	Run3	Run1	Run2	Run3
g_{21}	-0.01	-0.17	-0.24	-0.48	-0.68	-0.76
a_{22}	0.05	0.03	0.04	-0.42	-0.45	-0.43
t_{23}	-0.30	-0.31	-0.28	-0.79	-0.80	-0.79
t_{24}	-0.27	-0.20	-0.30	-0.78	-0.79	-0.82
g_{25}	-0.26	-0.18	-0.18	-0.81	-0.76	-0.75
g_{26}	-0.24	-0.17	-0.15	-0.74	-0.68	-0.69
t_{27}	-0.27	-0.28	-0.30	-0.81	-0.79	-0.83
g_{28}	-0.29	-0.22	-0.23	-0.79	-0.79	-0.78
c_{29}	-0.16	-0.10	-0.10	-0.71	-0.68	-0.61
t_{30}	-0.24	-0.26	-0.19	-0.81	-0.81	-0.78
**2**						
g_{15}	-0.21	-0.32	-0.16	-0.69	-0.85	-0.78
a_{16}	0.03	0.01	-0.02	-0.56	-0.49	-0.52
t_{17}	-0.37	-0.35	-0.37	-0.84	-0.82	-0.85
t_{18}	-0.17	-0.23	-0.20	-0.68	-0.76	-0.69

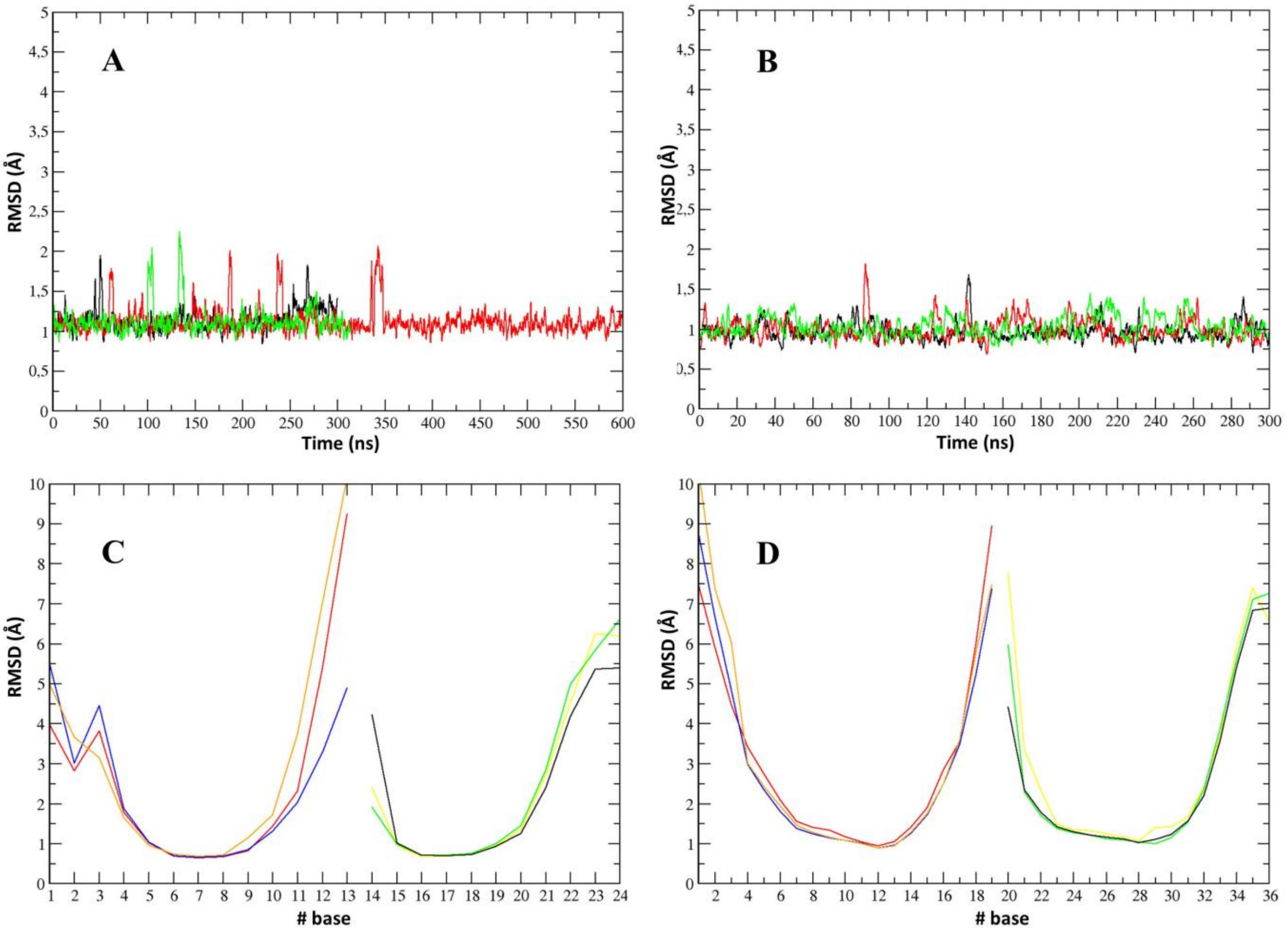

Figure S1. Up: Root-mean-square deviation versus time in the three MD runs on **2**/RNA (A) and **1**/RNA (B). Superpositions were made on the central base-pairs using thermalized structures as reference. Down: Root-mean-square fluctuations in the three MD runs on **2**/RNA (C) and **1**/RNA (D) where the X-axis represents the residue numbering in the sequence (see Table S1 for residue numbering). Colour codes: PNA strand run 1: yellow; RNA strand run 1: red; PNA strand run 2: green; RNA strand run 2: blue; PNA strand run 3: black; RNA strand run 3: orange.

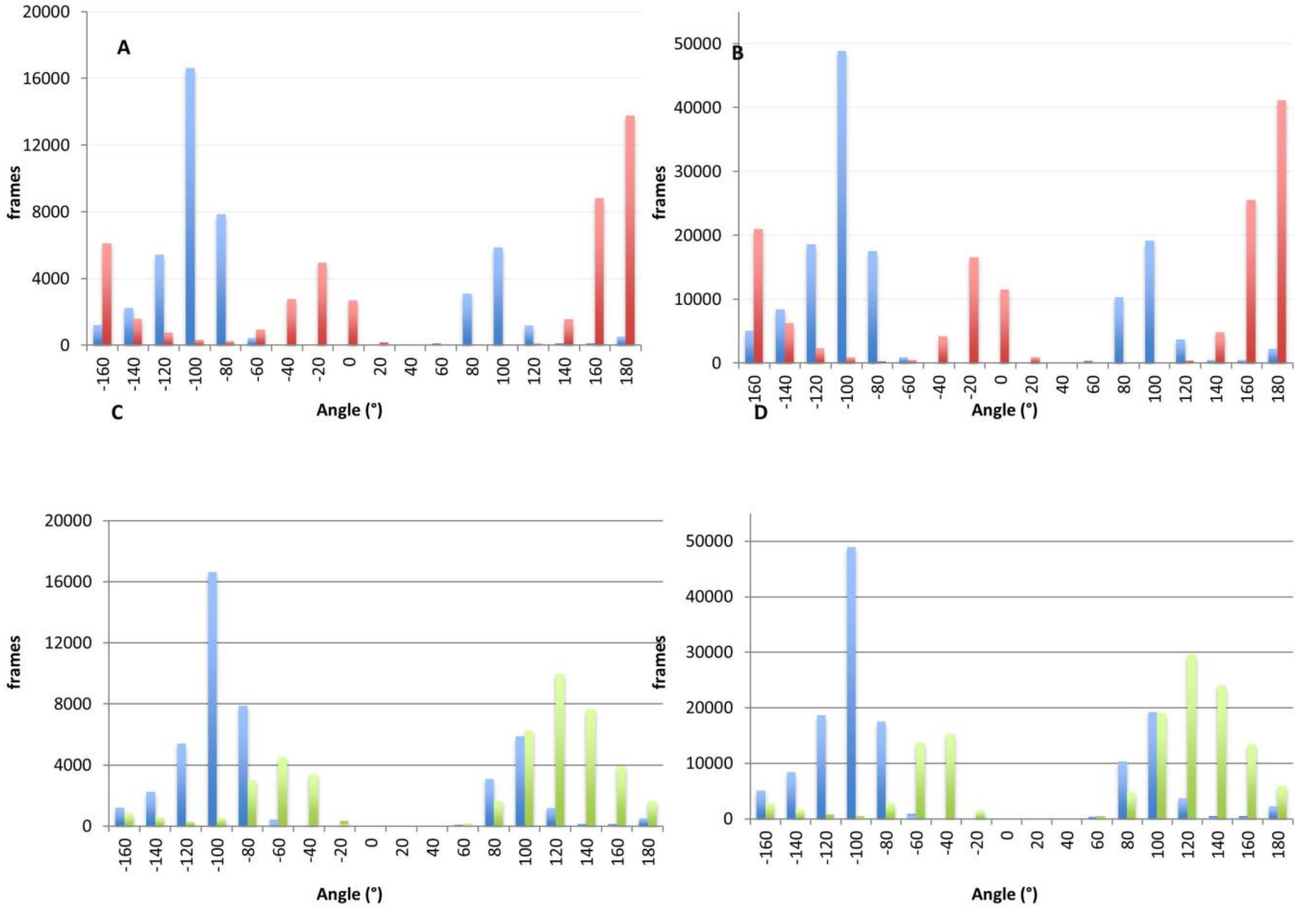

Figure S2. Distribution of the torsion angles α (blue) and ε (red) and the pseudo torsion angle ν (green) of PNA strand during the MD run of **2** (A and C) and **1** (B and D) heteroduplexes. Angles were sampled every 0.01 ns.
